# Molecular Dynamics Simulations to Investigate the Binding Mode of the Natural Product Liphagal with Phosphoinositide 3-Kinase α

**DOI:** 10.3390/molecules21070857

**Published:** 2016-06-29

**Authors:** Yanjuan Gao, Ying Ma, Guangde Yang, Yiping Li

**Affiliations:** School of Pharmacy, Xi’an Jiaotong University, No. 76 Yanta West Road, Xi’an 710061, China; gaoyanjuan@stu.xjtu.edu.cn (Y.G.); my417815@stu.xjtu.edu.cn (Y.M.); jmw52@mail.xjtu.edu.cn (G.Y.)

**Keywords:** molecular dynamics simulation, liphagal, phosphatidylinositol 3-kinase α, binding mode, anti-tumor

## Abstract

Phosphatidylinositol 3-kinase α (PI3Kα) is an attractive target for anticancer drug design. Liphagal, isolated from the marine sponge *Aka coralliphaga*, possesses the special “liphagane” meroterpenoid carbon skeleton and has been demonstrated as a PI3Kα inhibitor. Molecular docking and molecular dynamics simulations were performed to explore the dynamic behaviors of PI3Kα binding with liphagal, and free energy calculations and energy decomposition analysis were carried out by use of molecular mechanics/Poisson-Boltzmann (generalized Born) surface area (MM/PB(GB)SA) methods. The results reveal that the heteroatom rich aromatic D-ring of liphagal extends towards the polar region of the binding site, and the D-ring 15-hydroxyl and 16-hydroxyl form three hydrogen bonds with Asp810 and Tyr836. The cyclohexyl A-ring projects up into the upper pocket of the lipophilic region, and the hydrophobic/van der Waals interactions with the residues Met772, Trp780, Ile800, Ile848, Val850, Met922, Phe930, Ile932 could be the key interactions for the affinity of liphagal to PI3Kα. Thus, a new strategy for the rational design of more potent analogs of liphagal against PI3Kα is provided. Our proposed PI3Kα/liphagal binding mode would be beneficial for the discovery of new active analogs of liphagal against PI3Kα.

## 1. Introduction

The phosphoinositide 3-kinases (PI3Ks) are attractive targets for the design of small molecule inhibitors because of the frequent occurrence of aberrant signaling of this pathway in several different disease states such as tumor, inflammatory and autoimmune diseases [1,2]. Within the PI3 kinase family, there are four class I PI3 kinase isoforms (α, β, δ, and γ) [3]. PI3K pathway is one of the most commonly activated signaling pathways in cancer [4,5]. Especially, the PI3Kα isoform was found to be over-activated by mutation or loss of expression of the pathway suppressor phosphatase and tensin homologue deleted on chromosome 10 (PTEN) in colon, gastric, and breast carcinomas [6] and is likely to be the most commonly mutated kinase in the human genome [4].

The first generation PI3K inhibitors, wortmannin and LY294002 that is a synthetic analogue of the flavanoid quercetin, are derived from natural products, which have been widely employed as chemical genetics probes to elucidate the biological roles of PI3K signaling [7]. Other natural product inhibitors include myricetin, resveratrol, staurosporine and viridian [1].

**Figure 1:**
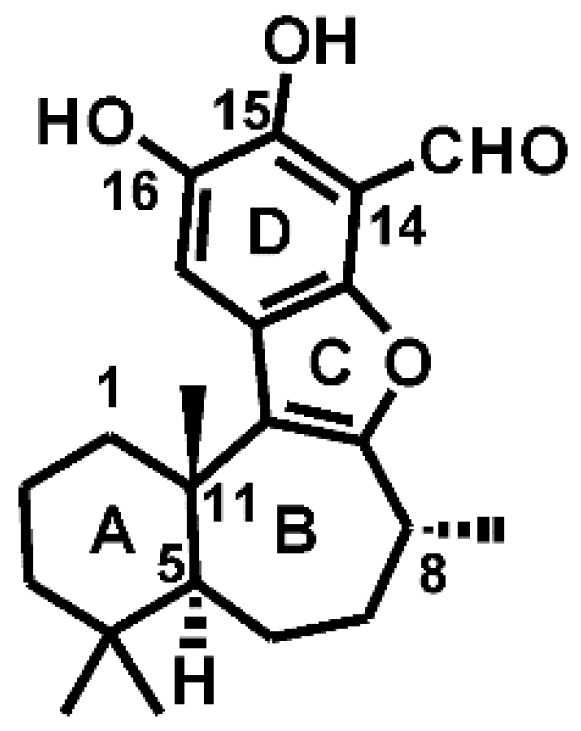
Chemical structure of liphagal.

Liphagal, as shown in Figure 1, is a tetracyclic meroterpenoid natural product isolated by Andersen et al. in 2006 during a program designed to discover new inhibitors of PI3K signaling pathway [8]. A crude methanol extract of the sponge *Aka coralliphaga* collected in Dominica showed promising activity, then bioassay-guided fractionation of the extract identified liphagal as the active component [8]. In this context, liphagal was found to have an IC_50_ value of 100 nM against PI3Kα and was tenfold more potent against PI3Kα than PI3Kγ. In addition, liphagal is cytotoxic to human cell line, such as LoVo, CaCo, and MDA-46 cell lines, with IC_50_ values of 0.58, 0.67, and 1.58 μM, respectively [8]. In 2010, Andersen et al. synthesized a small library of 12 liphagal analogues and identified a analogue with IC_50_ values of 66 nM against PI3Kα and 1840 nM against PI3Kγ, which exhibits modestly enhanced potency and isoform selectivity compared with the lead compound liphagal and also illustrates that liphagal is a useful starting point for the development of new PI3Kα inhibitors that might represent promising drug candidates and/or cell biology tools [9].

A key aspect of the inhibitor discovery process is to determinate the three-dimensional structure of the inhibitor-protein complex. However up to now there is not a three-dimensional structure of liphagal-PI3Kα complex available. Therefore, elucidating the binding mode of liphagal with PI3Kα could provide some clues to the design of more promising PI3Kα inhibitors. Several molecular dynamics simulations studies investigating the mechanism of PI3Kα overactivation by oncogenic mutations [10,11], potential allosteric modulation of PI3Kα [12], and isoform-specific inhibition of PI3Ks [13,14] have been published. In this study, molecular docking, molecular dynamics simulations and molecular mechanics/Poisson-Boltzmann (generalized Born) surface area (MM/PB(GB)SA) methods were applied as a powerfully computational strategy to investigate the detailed interactions of liphagal with PI3Kα. Finally, according to the binding mode of liphagal with PI3Kα identified in our work, a strategy for the design of more potent analogs of liphagal against PI3Kα is provided, which is helpful for further rational design of new inhibitor against PI3Kα.

## 2. Methods

### 2.1. Preparation of PI3Kα and Liphagal

The X-ray crystal structures of PI3Kα (PDB code 2RD0) was retrieved from the RCSB Protein Data Bank. The lost loop zones of the PI3Kα X-ray structure were generated and refined by ab initio refinement of the loop in the loop refine module of Modeler 9v5 [15]. The overall 2RD0 was subsequently subjected to 500 iterations of energy minimization with backbone atoms being restrained using the OPLS2005 force field [16] in the MacroModel module in the Schrodinger software suite [17]. Liphagal was built using the Maestro Build panel and minimized by the MacroModel program using the OPLS2005 force field.

### 2.2. Docking Experiments

First, the Gasteiger charges [18] for PI3Kα and liphagal prepared by the above method were calculated using AutoDock-Tools-1.5.4 [19]. Then their non-polar hydrogens were merged so that these hydrogen structures were not considered in the docking calculations. And the rotatable bonds of liphagal were set up. Second, energy affinity maps for atom types, desolvation energies, and electrostatic potentials of liphagal were pre-calculated using AutoGrid4. Third, the binding site on PI3Kα was defined by a grid system of (x, y, z) = (46-point, 46-point, 52-point) with a grid Spacing of 0.375 Å that originated at the center of the catalytic kinase domains by using the residue Val851 as the center of grid box. Finally, docking simulations were carried out via Autodock4 [20] with a rigid receptor structure, which allowed for flexibility in the ligand structure using a Lamarckian Genetic Algorithm (LGA) in combination with a hybrid local and global search for new docking conformations. The Lamarckian genetic algorithm was applied to the following protocol: trials of 100 runs, energy evaluations of 50,000,000, maximum number of generations of 30,000, population size of 200, a mutation rate of 0.02, a crossover rate of 0.8, and an elitism value of 1. The docking results were evaluated by sorting the docking energy predicted by docking conformations. Docked conformations were clustered using a tolerance of 2.0 Å root-mean-square deviations (rmsd).

### 2.3. MD Simulations of the PI3Kα/Liphagal Complex

The overall PI3Kα coordinate was concatenated with the docked coordinates of liphagal taken from the docking experiments. The atomic partial charges for liphagal were developed using Hartree-Fock/6-31 + G* calculations of the electrostatic potential with Gaussian03 suite [21], to which the RESP charges were fitted using the ANTECHAMBER [22] protocol of the Amber9 suit [23]. The atom types and the stretching, bending, dihedral, and improper dihedral parameters for liphagal were assigned based on the Generalized Amber Force Field (GAFF) [24], subsequently topology and parameter files were generated for liphagal.

All simulations were conducted by using the Amber9 program. Two parameter sets were used, the biomolecular force field ff03 [25] for the protein and general Amber Force Field (GAFF) for the organic small molecule. The PI3Kα/liphagal complex was soaked in a truncated octahedron box of TIP3P water molecules with a margin of 15 Å along each dimension. Nine Na^+^ ions were added to neutralize the system. The covalent bonds involving hydrogen atoms of the complex system were constrained using the SHAKE option [26], and the particle mesh Ewald (PME) method [27] was used to model the long-range electrostatic interactions using the parallel sander protocol on 16 cores of the IBM opteron cluster in National High Performance Computing Center (Xi’an). The system was then energy minimized with a 100 cycle steepest descent method, which was followed by a 1900 cycle conjugate gradient method. The temperature of the system was elevated from 100 K to 300 K over 50 ps via the Berendsen temperature coupling schemes in Amber using a TAUTP of 2.0 ps (time constant for heat bath coupling). The pressure of the system was equilibrated for 200 ps using the Berendsen pressure coupling schemes in Amber using a TAUP 2.0 ps (pressure relaxation time). Finally, a 10 ns production run was carried out and the trajectory of the complex structure was written out every 10 ps in order to collect 1000 snapshots.

### 2.4. Binding Free Energy Calculations

The binding free energies were calculated using the MM/PB(GB)SA method as implemented in Amber9. MM/PB(GB)SA computes the binding free energy by using a thermodynamic cycle that combines the molecular mechanical energies with the continuum solvent approaches [28]. The binding free energy was calculated according to the equation:

ΔG_bind_ = G_complex_ − G_PI3Kα_ − G_liphagal_ (1)

where G_complex_, G_PI3Kα_ and G_liphagal_ are the free energies of the complex, the protein PI3Kα and the ligand liphagal, respectively. The free energy of each term was calculated as a sum of the three terms:

G = E_MM_ + G_sol_ − TS (2)

where E_MM_ is the molecular mechanics energy of the molecule expressed as the sum of the internal energy (bonds, angles and dihedrals) (E_int_), electrostatic energy (E_ele_) and Van der waals term (E_vdw_) computed using an Amber99 force field:

E_MM_ = E_int_ + E_ele_ + E_vdw_ (3)

G_sol_ accounts for the solvation energy which can divided into the polar (G_PB(GB)_) and nonpolar part (G_NP_).

G_sol_ = G_PB(GB)_ + G_NP_ (4)

The polar part (G_PB(GB)_) accounts for the electrostatic contribution to solvation and was calculated using a Poisson-Boltzmann (PB) model and a Generalized-Boltzmann (GB) model at igb = 5 [29] via Amber9’s pbsa protocol [30] with a PARSE charge/radii set, a 1.4 Å solvent probe radius, and a 0.5 Å grid spacing. The solvent’s dielectric constant was set to 80, while the dielectric constant was set to 1 in the protein’s interior.

The nonpolar part (G_NP_) accounts for the nonpolar contribution to solvation and was approximated by relating it to the solvent accessible surface area (SASA) with coefficient of 0.0072 [31].

The entropy contribution (−TS) arising from changes in the degrees of freedom (translational, rotational, and vibrational) of the solute molecules was included applying classical statistical thermodynamics. Entropy contribution was calculated using an nmode protocol with a distance dependent dielectric constant [32].

After including all the energetic terms for PI3Kα, liphagal and the complex Equation (1) can be reorganisated and expressed as:

ΔG_bind_ = ΔE_MM_ + ΔG_sol_ − TΔS (5)

where ΔE_MM_, ΔG_sol_ and ΔS are simply the change in the internal energy, the solvation energy and the entropy between PI3Kα, liphagal and the complex. Binding free energy was calculated using 700 snapshots sampled with ptraj program every 10 ps; these snapshots cover the last 7 ns of the MD trajectory. Due to the high computational demand, the entropy calculations were performed only for every tenth one of the 700 snapshots (70 snapshots in total) described above.

### 2.5. Free Energy Decomposition

In order to identify the residues that contribute the most to the calculated overall binding energy, we used a residue-by-residue decomposition protocol embedded in the GB solvent model based in MMGBSA. The GB model is an alternative to the PB solvation model that uses a pair-wise analytical approximation of the PB model. Using this model the calculated energies can be further broken down into individual residue’s contributions. The decomposition was performed only for molecular mechanics and salvation energies but not for entropies. The binding interaction of liphagal-residue pair includes four terms: van der Waals contribution, electrostatic contribution, polar solvation contribution, and nonpolar solvation contribution.

## 3. Results and Discussion

### 3.1. Docking Liphagal to the Crystal Structure of PI3Kα

**Figure 2:**
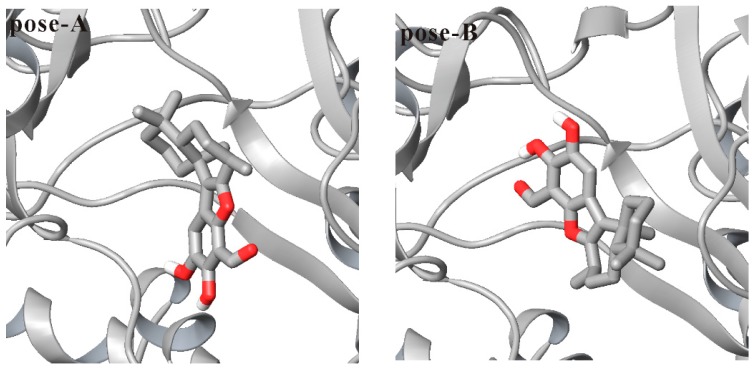
Two docked conformations of liphagal with the X-ray crystal structures of PI3Kα (pose-A: liphagal of pose-A/PI3Kα; pose-B: liphagal of pose-B/PI3Kα; PI3Kα (silver) is shown as cartoon, while liphagal is shown as tubes with silver carbon and red oxygen).

Because no liphagal-bound PI3Kα crystal has been solved, liphagal was docked into the PI3Kα ATP-binding site (PDB code 2RD0) to obtain the liphagal-bound complex for further molecular dynamics simulations. One hundred docked conformations of liphagal for PI3Kα obtained in our molecular docking experiment were clustered to 5 clusters using a tolerance of 2.0 Å rmsd. The lowest docking energy among 100 docked conformations is −8.31 kcal·mol^−1^, and this cluster includes 35 docked conformations. The ranked second cluster has 46 conformations, and its lowest docking energy among 46 conformations is −7.73 kcal·mol^−1^. The two poses of liphagal with PI3Kα, named as pose-A and pose-B respectively, are shown in Figure 2. As seen from Figure 2, the orientations of pose-A and pose-B are very different, the heteroatom rich aromatic D-ring of pose-A extends towards the polar region of the binding site in PI3Kα, while the D-ring of pose-B extends towards the hinge region. The orientation of pose-A is the lowest docking energy conformations, and is consistent with that of liphagal with PI3Kγ, which was obtained only using Surflex-Dock as implemented by Sybyl 7.2 [33], but the orientation of pose-B is the lowest docking energy conformations included in the largest cluster. The lowest docking energy conformations or the lowest docking energy conformations included in the largest cluster are considered to be the most stable orientations. Therefore, both pose-A and pose-B were selected as the initial conformation of liphagal for molecular dynamics simulation to get more reasonable binding mode of liphagal with PI3Kα, where the flexibility of receptor is considered.

### 3.2. Molecular Dynamics Simulation of Liphagal-Bound PI3Kα

**Figure 3:**
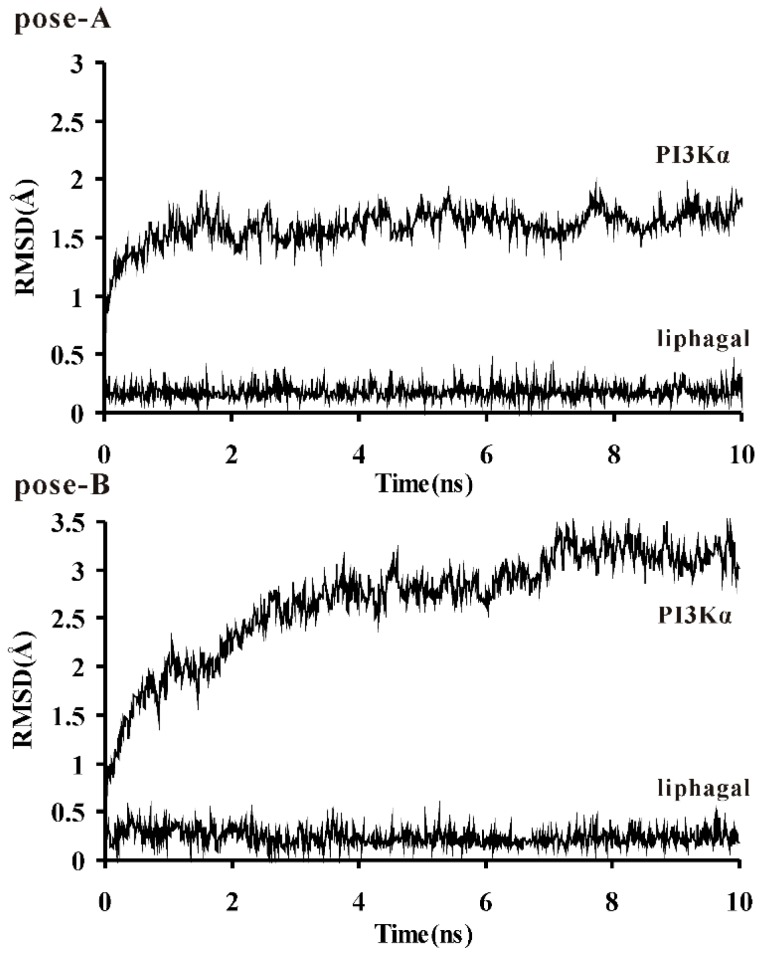
RMSD of the backbone atoms of the catalytic kinase domain of PI3Kα and the heavy atoms of liphagal (pose-A: liphagal of pose-A/PI3Kα; pose-B: liphagal of pose-B/PI3Kα).

To explore the dynamic stability of these two protein/inhibitor complexes and to ensure the rationality of the sampling strategy, the backbone atoms root-mean-square deviation (rmsd) of PI3Kα catalytic kinase domain and the heavy atoms rmsd of liphagal were calculated based on the starting snapshot and plotted in Figure 3. The rmsd plots indicate that the conformations of PI3Kα of pose-A achieve equilibrium around 1.0 ns and fluctuate around 1.5 Å, while for the PI3Kα of pose-B, the equilibrium time is around 3.0 ns and the conformations fluctuate around 3.0 Å. The rmsds of liphagal of pose-A and pose-B are stable in the simulation process. Both trajectories are stable after 3.0 ns, so the snapshots extracted from 3.0 to 10.0 ns were used to the binding free energy calculation and free energy decomposition.

The calculated binding free energies and individual energy components are listed in Table 1. As what suggests in Table 1, for pose-A, the contributions of the molecular mechanics part (ΔE_MM_) and the solvation part (ΔG_pb_sol_, ΔG_gb_sol_) are calculated to be −87.80 kcal·mol^−1^, 53.19 kcal·mol^−1^ and 49.30 kcal·mol^−1^, respectively. According to the equation ΔG_bind_ = ΔE_MM_ + ΔG_sol_ − TΔS, adding the entropy contribution (TΔS, −24.13 kcal·mol^−1^) calculated by nmode protocol, the binding free energy (ΔG_bind_) between PI3Kα and liphagal of pose-A is −10.48 kcal·mol^−1^ using MMPBSA method, while −14.37 kcal·mol^−1^ using MMGBSA method, which is beneficial for binding. However, for pose-B, the binding free energy is −1.06 kcal·mol^−1^ using MMPBSA method, while −8.22 kcal·mol^−1^ using MMGBSA method, which is beneficial for binding too. Thus, these two PI3Kα and liphagal complexes formations exemplify a classical favorable reaction in solution where the increase of the stability produced by the formation of the complex overcomes the cost of the entropy and desolvation of protein and ligand. The molecular mechanics energy favors the PI3Kα/liphagal complex formation, while the salvation energy and the entropy disfavor the complex formation, and the molecular mechanics energy makes the prominent contribution to the binding energy, which drives the complex formation. Notably, the binding free energies of the PI3Kα/liphagal of pose-A complex are lower than those of the PI3Kα/liphagal of pose-B, respectively, which suggests that pose-A is the favorable binding mode. ΔG_bind_ between PI3Kα and liphagal is −9.93 kcal·mol^−1^, which was calculated by the formula ΔG = RT lnK_i_ using the experimental IC_50_ value of liphagal for PI3Kα (IC_50_ = 0.1 μM). According to the Cheng-Prusoff equation, K_i_ = IC_50_/(1 + [S]/K_m_), K_i_ is less than or equal to IC_50_. So ΔG should be less than or equal to −9.93 kcal·mol^−1^. From this point, ΔG_bind_ between PI3Kα and liphagal of pose-A by MM/PB(GB)SA is good agreement with the experimental IC_50_ value of liphagal for PI3Kα (IC_50_ = 0.1 μM).

In order to get a better view on which energy term has more impact on the binding affinity of the complexes, the four individual energy components (ΔE_vdw_, ΔE_ele_, ΔG_pb(gb)_ and ΔG_pb(gb)_sur_) were carefully compared. The ΔE_ele_ of the PI3Kα/liphagal of pose-A complex (−50.06 kcal·mol^−1^) is significantly stronger than that of the PI3Kα/liphagal of pose-B complex (−7.51 kcal·mol^−1^), while the ΔE_vdw_ of the PI3Kα/liphagal of pose-A complex (−37.74 kcal·mol^−1^) is almost as same as that of the PI3Kα/liphagal of pose-B complex (−37.96 kcal·mol^−1^). The ΔG_pb_ and ΔG_gb_ of the PI3Kα/liphagal of pose-A complex (58.84 kcal·mol^−1^, 54.95 kcal·mol^−1^) are weaker than those of the PI3Kα/liphagal of pose-B complex (29.82 kcal·mol^−1^, 22.66 kcal·mol^−1^), while the ΔG_pb_sur_ and ΔG_gb_sur_ of the PI3Kα/liphagal of pose-A complex (−5.65 kcal·mol^−1^, −5.65 kcal·mol^−1^) are almost as same as those of the PI3Kα/liphagal of pose-B complex (−5.94 kcal·mol^−1^, −5.94 kcal·mol^−1^). Considering the polar contribution of desolvation (ΔG_pb(gb)_), the net electrostatic contributions (ΔE_ele_ +ΔG_pb(gb)_) of the PI3Kα/liphagal of pose-A and the PI3Kα/liphagal of pose-B complexes are 8.78 and 22.31 kcal·mol^−1^ using PB model, respectively, while 4.89 and 15.15 kcal·mol^−1^ using GB model, which suggests that although the electrostatic contribution encourages the binding, it still cannot fully cover the negative effect produced by the polar contribution of desolvation, thus the net electrostatic contribution disfavors complex formation. And considering the non-polar contribution of desolvation (ΔG_pb(gb)_sur_), the total hydrophobic interaction contributions (ΔE_vdw_ +ΔG_pb(gb)_sur_) of the PI3Kα/liphagal of pose-A and the PI3Kα/liphagal of pose-B complexes are −43.39 and −43.9 kcal·mol^−1^ using PB and GB model, respectively, and thus favor complex formation. Furthermore, the difference values of the electrostatic contribution between the PI3Kα/liphagal of pose-A and the PI3Kα/liphagal of pose-B complexes are −13.53 and −10.26 kcal·mol^−1^ using PB and GB model, respectively, and the difference values of the hydrophobic interaction contribution between them are both 0.51 kcal·mol^−1^ using PB and GB model. Thus, the electrostatic contribution results in the lower binding free energies of the PI3Kα/liphagal of pose-A complex than those of the PI3Kα/liphagal of pose-B, and plays a key role in differentiating these two conformations of liphagal.

As well known, hydrogen bond is an important interaction in protein-ligand complex formation. However, in MM/PB(GB)SA method, hydrogen bond contribution is included to electrostatic interaction, which is not explicitly calculated. So to further investigate electrostatic interaction, hydrogen bond interactions between PI3Kα and liphagal of pose-A and pose-B were clustered based on liphagal of pose-A and pose-B, see Table 2. As seen from Table 2, these two conformations of liphagal lead to some different hydrogen bonding interactions. Liphagal of pose-A can form three very stable hydrogen bonds, which are between the D-ring 15-hydroxyl of liphagal and the side chain carboxyl oxygen of Asp810 in PI3Kα, and the D-ring 16-hydroxyl of liphagal and the side chain carboxyl oxygen of Asp810 and the side chain hydroxyl of Tyr836, respectively. However, liphagal of pose-B can form only one hydrogen bond, which is between the D-ring 14-formyl oxygen of liphagal and the backbone NH of Val851 in PI3Kα and is less stable than hydrogen bonding interactions of liphagal of pose-A with PI3Kα. Therefore, the difference of hydrogen bonding interactions between the PI3Kα/liphagal of pose-A and the PI3Kα/liphagal of pose-B complexes accounts for the difference values of the electrostatic contribution between them.

### 3.3. Decomposition of Binding Energy on a Per-Residue Basis

**Figure 4:**
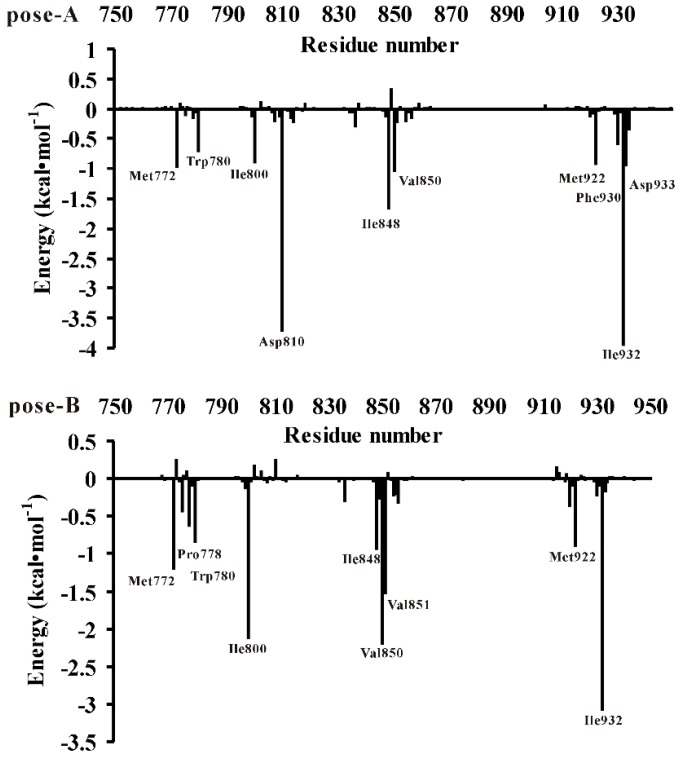
Decomposition of the binding enthalpy (ΔE_MM_ + ΔG_sol_) on a per-residue for residues of the catalytic kinase domains of PI3Kα (pose-A: liphagal of pose-A/PI3Kα; pose-B: liphagal of pose-B/PI3Kα).

For the purpose of obtaining the detailed presentation of the liphagal of pose-A and pose-B/PI3Kα interactions, the MM/GBSA binding energy (the binding enthalpy) decomposition analysis was employed to decompose the total binding energies into the residues of PI3Kα. The quantitative information of each residue’s contribution is extremely useful to discern the difference of the binding mode of the liphagal of pose-A and pose-B with PI3Kα. The contributions of each residue of PI3Kα binding site were plotted in Figure 4. Figure 4 shows the binding affinity of liphagal of pose-A mainly depends on residues Met772, Trp780, Ile800, Asp810, Ile848, Val850, Met922, Phe930, Ile932 and Asp933. On the other hand, from Figure 4, liphagal of pose-B has strong interactions with residues Met772, Pro778, Trp780, Ile800, Ile848, Val850, Val851, Met922 and Ile932. The comparison of two figures indicates liphagal of pose-A and pose-B have similar interactions with residues Met772, Pro778, Trp780, Met922 and Phe930, which also agrees with “hot-spot” residues of PI3Kα/wortmannin complex [13]. However, liphagal of pose-A and pose-B differently interact with residues Ile800, Asp810, Ile848, Val850, Val851, Ile932 and Asp933. Especially, Asp810 and Val851 are the key residues for the distinction between liphagal of pose-A (−3.70 and −0.23 kcal·mol^−1^) and pose-B (0.26 and −1.52 kcal·mol^−1^), which can form hydrogen bond with liphagal of pose-A and pose-B, respectively. And other residues have different hydrophobic/van der Waals interactions with liphagal pose-A and pose-B.

### 3.4. Dynamics Analysis of the Interactions between PI3Kα and Liphagal

**Figure 5:**
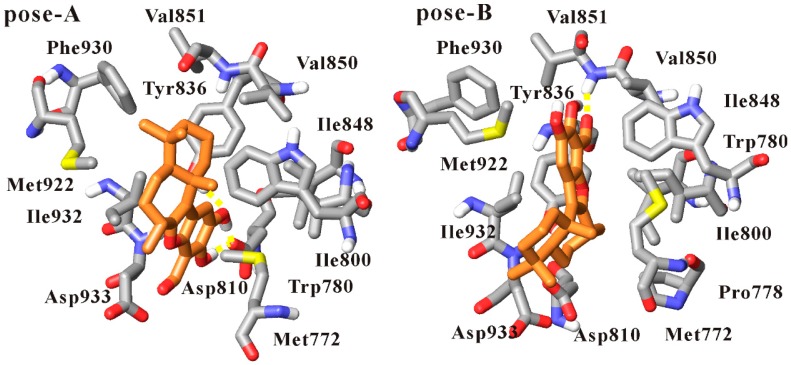
The representative structures from molecular dynamics simulation of liphagal-bound PI3Kα (pose-A: liphagal of pose-A/PI3Kα; pose-B: liphagal of pose-B/PI3Kα; liphagal and the amino acids interacted with liphagal are shown as tubes with orange (liphagal) and silver (the amino acids) carbon, red oxygen, blue nitrogen and yellow sulfur atoms. Hydrogen bonds are shown as yellow dashes).

To further understand the different binding modes of liphagal of pose-A and pose-B, the trajectory files of 10 ns molecular dynamics simulation of liphagal of pose-A and pose-B with PI3Kα were clustered by the average-linkage clustering algorithm, and these two trajectory files were clustered to only one cluster, respectively. The representative structures were extracted from these two clusters, respectively, shown in Figure 5. (The movies produced by using the trajectory files of 10 ns molecular dynamics simulation of liphagal of pose-A and pose-B with PI3Kα were included in the Supplementary Materials).

In the simulation of liphagal of pose-A-bound PI3Kα, as seen from Figure 5, the conformation of liphagal of pose-A keeps stable within 10 ns simulation. The (6-7-5-6) tetracyclic skeleton of liphagal inserts deeply into the binding site of PI3Kα, and accommodates with this site. The heteroatom rich aromatic D-ring extends towards the polar region of the binding site, and the D-ring 15-hydroxyl and 16-hydroxyl can form three hydrogen bonds with the side chain carboxyl oxygen of Asp810 and the side chain hydroxyl of Tyr836. The cyclohexyl A-ring projects up from the aromatic plane and into the upper pocket of the lipophilic region, which coincides with the adenine-binding region. Liphagal forms hydrophobic/van der Waals interactions with the residues Met772, Trp780, Ile800, Ile848, Val850, Val851, Met922, Phe930, Ile932, Asp933.

As what suggests in Figure 5, the conformation of liphagal of pose-B is stable within 10 ns simulation, and liphagal extends deeply into the binding site of PI3Kα. However, compared with liphagal of pose-A, the D-ring extends towards the hinge region, and the D-ring 14-formyl oxygen can form one hydrogen bond with the backbone NH of Val851. Liphagal forms hydrophobic/van der Waals interactions with the lipophilic region of the binding site, characterized by the residues Met772, Pro778, Trp780, Ile800, Ile848, Val850, Val851, Met922 and Ile932.

Thus, from Figure 5, it can be observed that the binding modes of liphagal of pose-A and pose-B with PI3Kα are significantly different. The orientations of the (6-7-5-6) tetracyclic skeleton of liphagal of pose-A and pose-B are different, which results in the difference of the orientation and amount of hydrogen bonds and further the difference of the hydrophobic/van der Waals interactions. Asp810 and Tyr836 are the key residues to form hydrogen bonds with liphagal of pose-A, while Val851 is the key residue for liphagal of pose-B. Especially, liphagal of pose-A is close to the polar region of the binding site, while liphagal of pose-B is close to the hinge region, thus causing that the van der Waals interactions between liphagal of pose-A and the residues Ile848, Ile932 and Asp933 are stronger than those between liphagal of pose-B and those residues, and the van der Waals interactions between liphagal of pose-B and the residues Ile800 and Val850 are stronger than those between liphagal of pose-A and those residues (Supplementary Materials Figure S1). These differences of the interactions can account for the different contributions of an identical residue to the bindings of liphagal of pose-A and pose-B with PI3Kα using the free energy decomposition method by residue above. Therefore, based on the binding free energies and the free energy decomposition analysis, pose-A is the favorable binding mode of liphagal with PI3Kα.

To clear the selectivity of liphagal to PI3Kα against PI3Kγ, the amino acids of PI3Kα that can interact with liphagal were compared with those of PI3Kγ. But no non-conservative amino acid was found in these amino acids interacted with liphagal, suggesting that the selectivity of liphagal could not be concerned with amino acid sequence variation within its binding region. According to Sabbah’s study [14], Ser774 (PI3Kα)/Ser806 (PI3Kγ) as a conservative amino acid may play a critical role in PI3Kα/γ-isoform-specific binding. But the interaction between the residue Ser774 and liphagal is weak, which suggests that the residue Ser774 could not be related to the selectivity of liphagal to PI3Kα. In these amino acids, Met772, which is located within the p-loop in the catalytic domain of PI3Kα and forms the ceiling of the adenine-binding pocket, was identified as a conformationally mobile residue to be responsible for the selectivity of the inhibitor against PI3K isoforms [34]. From the MM/GBSA binding energy decomposition analysis by residue, the contribution of Met772 to the binding is −0.97 kcal·mol^−1^. Thus, it can be inferred that Met772 could be the key residue for the selectivity of liphagal to PI3Kα.

Therefore, more importantly, based on the binding mode of liphagal with PI3Kα identified in our work, a strategy for the design of more potent analog of liphagal against PI3Kα is provided. The 2-carbon atom of cyclohexyl A-ring could be transformed into a heteratom, such as O, N, or a heteratom could be introduced into the 2-cyclohexyl A-ring, which could form a hydrogen bond with the backbone NH of Val851, thus improve the potential (Supplementary Materials Figure S2). A bulky group could be introduced into the 8-B-ring, which is anticipated to form stronger van der Waals interaction with Met772 in the p-loop, thus increase the selectivity and further affinity. The retrosynthesis of these modifications were included in Supplementary Materials Schemes S1–S3.

## 4. Conclusions

The binding free energies of the PI3Kα/liphagal of pose-A complex by MM/PB(GB)SA methods are lower than those of the PI3Kα/liphagal of pose-B, respectively, which suggests that pose-A is the favorable binding mode. The heteroatom rich aromatic D-ring extends towards the polar region of the binding site, and the D-ring 15-hydroxyl and 16-hydroxyl form three hydrogen bonds with the side chain carboxyl oxygen of Asp810 and the side chain hydroxyl of Tyr836. The cyclohexyl A-ring projects up from the aromatic plane and into the upper pocket of the lipophilic region, which coincides with the adenine-binding region and forms hydrophobic/van der Waals interactions with the residues Met772, Trp780, Ile800, Ile848, Val850, Val851, Met922, Phe930, Ile932, Asp933. The binding mode of liphagal with PI3Kα presented in this work may be very helpful for the development of more potent compounds to target PI3Kα.

## Figures and Tables

**Table 1:** Energy components and binding free energies for the PI3Kα/liphagal of pose-A and pose-B complexes.

Energies	PI3Kα/Liphagal of Pose-A		PI3Kα/Liphagal of Pose-B	
	Mean (kcal·mol^−1^)	Std (kcal·mol^−1^)	Mean (kcal·mol^−1^)	Std (kcal·mol^−1^)
ΔE_ele_	−50.06	4.05	−7.51	1.81
ΔE_vdw_	−37.74	3.58	−37.96	2.31
ΔE_MM_	−87.80	3.86	−45.47	2.57
ΔG_pb_sur_	−5.65	0.22	−5.94	0.22
ΔG_pb_	58.84	3.05	29.82	3.55
ΔG_pb_sol_	53.19	3.02	23.88	3.47
ΔG_gb_sur_	−5.65	0.22	−5.94	0.22
ΔG_gb_	54.95	2.44	22.66	1.71
ΔG_gb_sol_	49.30	2.43	16.72	1.64
ΔH_pb_	−34.61	3.61	−21.59	3.06
ΔH_gb_	−38.50	2.96	−28.75	2.03
TΔS	−24.13	16.18	−20.53	15.09
ΔG_bind(pb)_	−10.48		−1.06	
ΔG_bind(gb)_	−14.37		−8.22	

ΔE_ele_, electrostatic contribution; ΔE_vdw_, van der Waals contribution; ΔE_MM_, molecular mechanics contribution, ΔE_MM_ = ΔE_ele_ + ΔE_vdw_; ΔG_pb_sur_, the nonpolar contribution of desolvation by PBSA; ΔG_pb_, the polar contribution of desolvation by PBSA; ΔG_pb_sol_, the contribution of desolvation by PBSA, ΔG_pb_sol_ = ΔG_pb_sur_ + ΔG_pb_; ΔG_gb_sur_, the nonpolar contribution of desolvation by GBSA; ΔG_gb_, the polar contribution of desolvation by GBSA; ΔG_gb_sol_, the contribution of desolvation by GBSA, ΔG_gb_sol_ = ΔG_gb_sur_ + ΔG_gb_; ΔH_pb_ = ΔE_MM_ + ΔG_pb_sol_; ΔH_gb_ = ΔE_MM_ + ΔG_gb_sol_; TΔS, the entropy at temperature T; ΔG_bind(pb)_ = ΔH_pb_ − TΔS; ΔG_bind(gb)_ = ΔH_gb_ – TΔS.

**Table 2:** Hydrogen bonds of all of trajectories.

Inhibitor	Hydrogen Bond		Occupancy (%)	Distance (Å)
	Liphagal of Pose-A	PI3Kα		
Liphagal of pose-A	benzofuran ring 15-OH	*O*-Asp810	89.4	2.59 (0.09)
	benzofuran ring 16-OH	*O*-Asp810	87.4	2.59 (0.09)
	benzofuran ring 16-*O*	OH-Tyr836	97.2	2.85 (0.15)
Liphagal of pose-B	Liphagal of pose-B	PI3Kα		
	benzofuran ring 14-formyl-*O*	NH-Val851	70.0	3.05 (0.26)

Hydrogen bonds were defined by acceptor-donor atom distances of <3.2 Å and acceptor-H-donor angles of >120°. Hydrogen bonds are reported only if they exist for >10% of the investigated time period. Occupancy is in units of percentage of the investigated time period.

## Supplementary Materials

Supplementary materials can be accessed at: http://www.mdpi.com/1420-3049/21/7/857/s1.
