# Supplementary material for: Molecular Dynamics Simulations to Investigate the Binding Mode of the Natural Product Liphagal with Phosphoinositide 3-Kinase α
Source: Molecules. 2016 Jun 29;21(7):857. doi: 10.3390/molecules21070857 (PMC6274547; doi:10.3390/molecules21070857)
Supplement: Supplementary file 1 [file molecules-21-00857-s001.pdf]

Supplementary Materials: Molecular Dynamics Simulations to Investigate the Binding Mode of the Natural Product Liphagal with Phosphoinositide 3-Kinase *α*

Yanjuan Gao, Ying Ma, Guangde Yang and Yiping Li

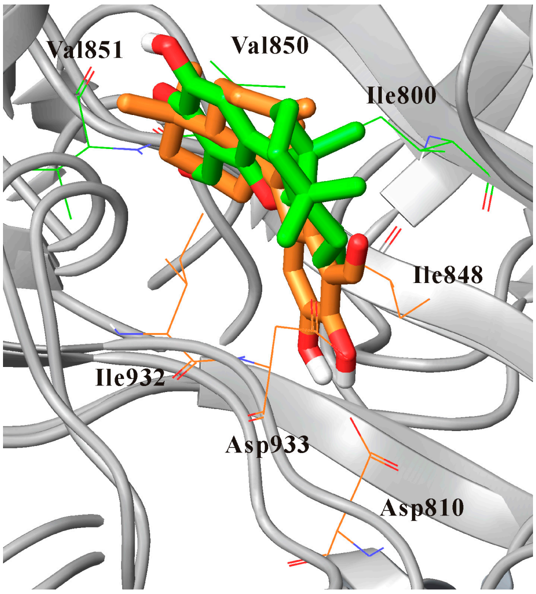

Figure S1. The superimposition of the catalytic kinase domains of liphagal-bound PI3K*α* (PI3K*α* (silver) is shown as cartoon, and liphagal of pose-A and pose-B are shown as tubes with orange and green carbon, respectively. The amino acids interacted with liphagal of pose-A and pose-B are shown as wires with orange and green carbon, respectively, red oxygen, blue nitrogen).

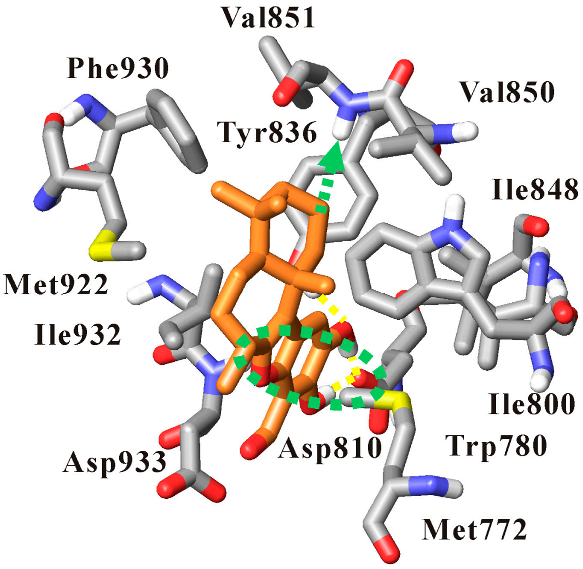

Figure S2. A strategy for the design of more potent analog of liphagal against PI3K*α* (liphagal and the amino acids interacted with liphagal are shown as tubes with orange (liphagal) and silver (the amino acids) carbon, red oxygen, blue nitrogen and yellow sulfur atoms. Hydrogen bonds are shown as yellow dashes. The green arrow represents hydrogen bond with NH of Val851, and the green oval represents the bulky group interacted with the side chain of Met772 by van der Waals interaction).

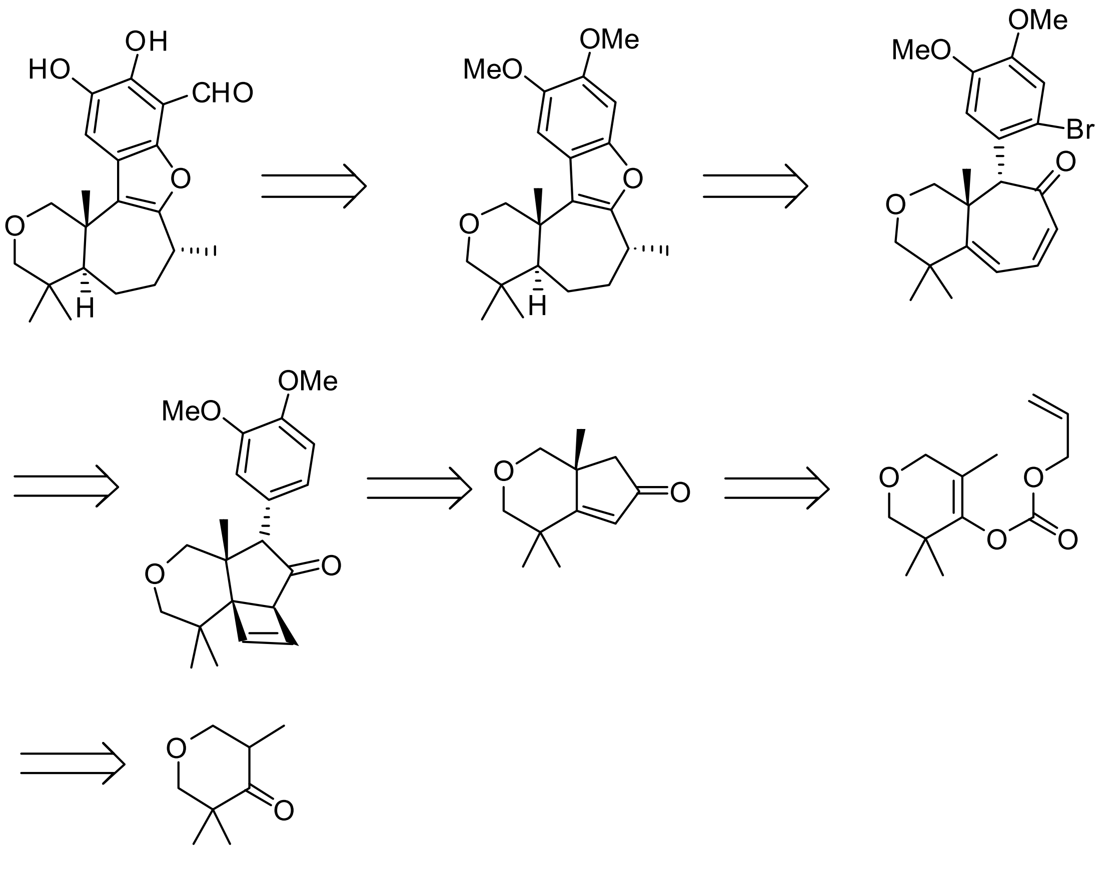

1556703-18-4

Scheme S1. The 2-carbon atom of cyclohexyl A-ring is transformed into oxygen atom [1].

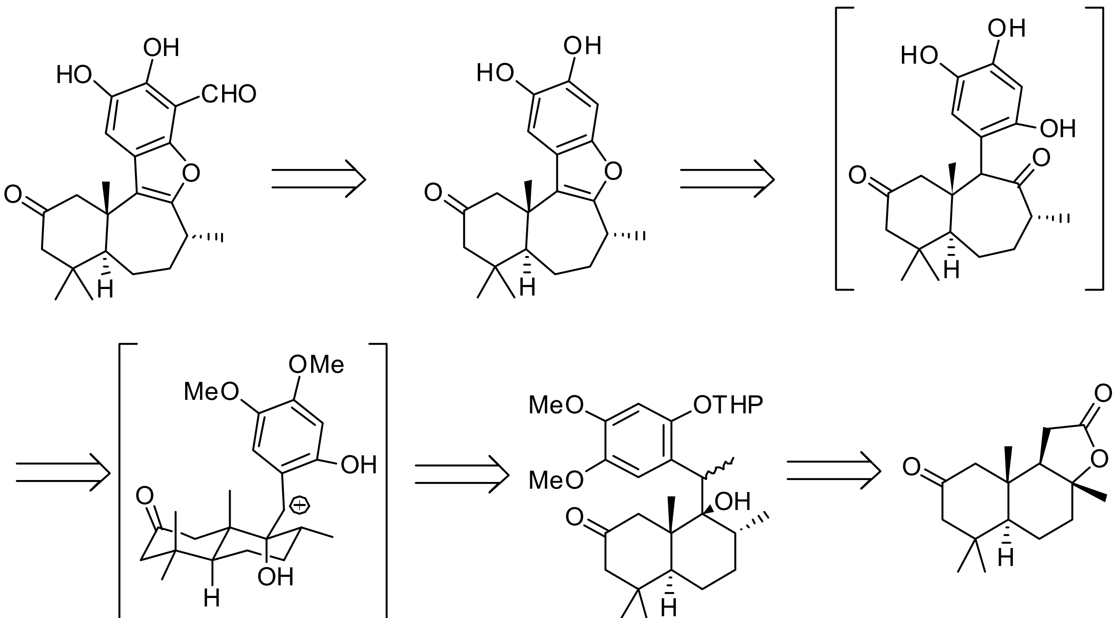

29065-00-7

Scheme S2. Oxygen atom is introduced into the 2-cyclohexyl A-ring [2].

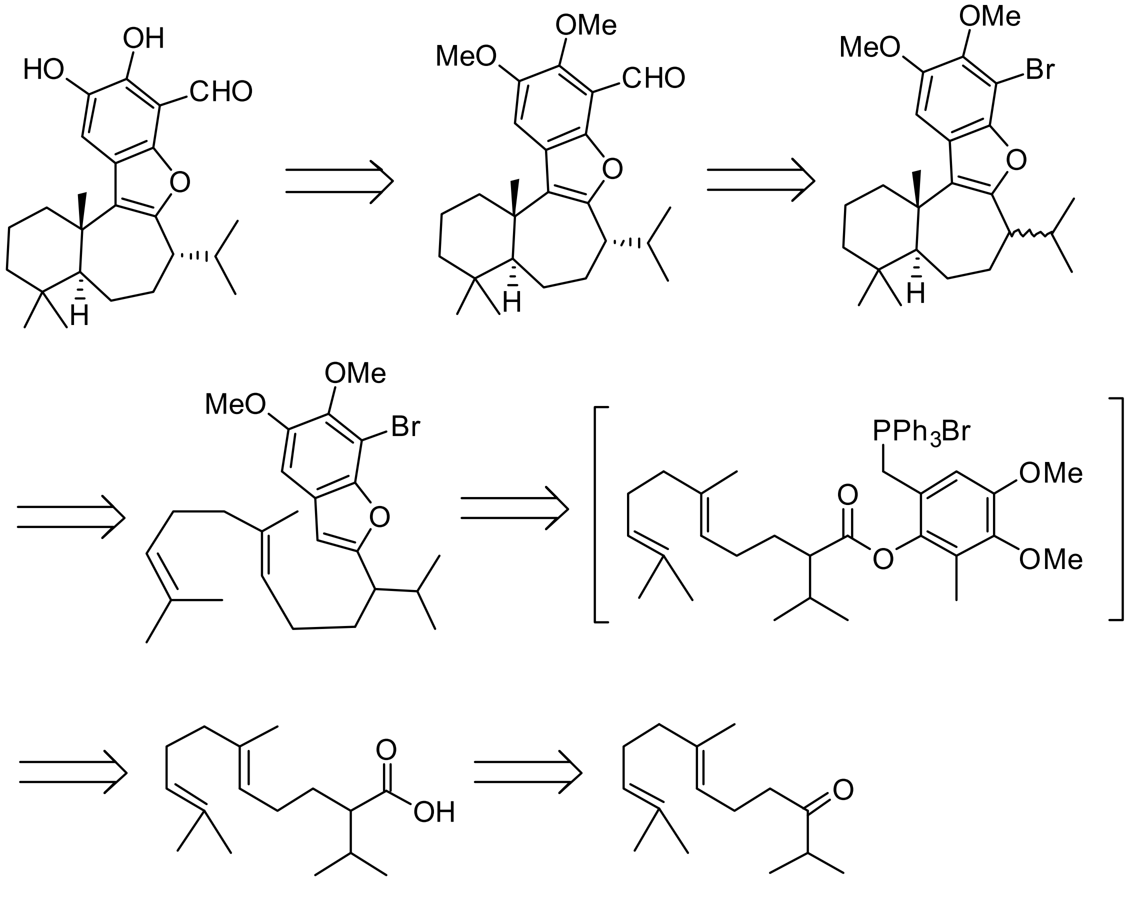

Scheme S3. The isopropyl group is introduced into the 8-B-ring [3].

References

1 Day, J.J.; McFadden, R.M.; Virgil, S.C.; Kolding, H.; Alleva, J.L.; Stoltz, B.M. The catalytic enantioselective total synthesis of (+)-liphagal. *Angew. Chem. Int. Ed. Engl.* **2011**, *50*, 6814–6818.

2 George, J.H.; Baldwin, J.E.; Adlington, R.M. Enantiospecific, biosynthetically inspired formal total synthesis of (+)-liphagal. *Org. Lett.* **2010**, *12*, 2394–2397.

3 Marion, F.; Williams, D.E.; Patrick, B.O.; Hollander, I.; Mallon, R.; Kim, S.C.; Roll, D.M.; Feldberg, L.; van Soest, R.; Andersen, R.J. Liphagal, a Selective inhibitor of PI3 kinase alpha isolated from the sponge akacoralliphaga: structure elucidation and biomimetic synthesis. *Org Lett.* **2006**, *8*, 321–324.
